# Quantitative Tyrosine Phosphoproteome Profiling of AXL Receptor Tyrosine Kinase Signaling Network

**DOI:** 10.3390/cancers13164234

**Published:** 2021-08-23

**Authors:** Xinyan Wu, Li Wang, Nicole A. Pearson, Santosh Renuse, Ran Cheng, Ye Liang, Dong-Gi Mun, Anil K. Madugundu, Yaoyu Xu, Parkash S. Gill, Akhilesh Pandey

**Affiliations:** 1 Department of Laboratory Medicine and Pathology, Mayo Clinic, Rochester, MN 55905, USA; Wang.Li3@mayo.edu (L.W.); renuse.santosh@mayo.edu (S.R.); chengran@bjmu.edu.cn (R.C.); Mun.Dong-Gi@mayo.edu (D.-G.M.); madugundu.anil@mayo.edu (A.K.M.); Yaoyu.xu@aynu.edu.cn (Y.X.); 2 Molecular Pharmacology and Experimental Therapeutics, Mayo Clinic, Rochester, MN 55905, USA; Pearson.Nicole1@mayo.edu; 3 Center for Individualized Medicine, Mayo Clinic, Rochester, MN 55905, USA; 4 Department of Biological Chemistry, Johns Hopkins University School of Medicine, Baltimore, MD 21205, USA; ye.liang@umassmed.edu; 5 Manipal Academy of Higher Education (MAHE), Manipal 576104, Karnataka, India; 6 Department of Medicine, Keck School of Medicine, University of Southern California, Los Angeles, CA 90089, USA; parkashg@med.usc.edu; 7 Center for Molecular Medicine, National Institute of Mental Health and Neurosciences (NIMHANS), Hosur Road, Bangalore 560029, Karnataka, India

**Keywords:** AXL, GAS6, receptor tyrosine kinase, protein tyrosine phosphatase, phosphorylation, signaling, proteomics, mass spectrometry, breast cancer

## Abstract

**Simple Summary:**

AXL is a receptor tyrosine kinase belonging to the TAM (Tyro3, Axl and Mer) family. The AXL protein plays an important role in promoting cancer development, such as proliferation, migration, invasion and survival of cancer cells. In this study, we used mass spectrometry-based proteomics to quantify the cancer signaling regulated by AXL activation. Our study identified more than 1000 phosphotyrosine sites and discovered that activation of AXL can upregulate multiple cancer-promoting and cell migration/invasion-related signaling pathways. We also observed significant crosstalk as evidenced by rapid phosphorylation of multiple receptor tyrosine kinases and protein tyrosine phosphatases, including PTPN11 and PTPRA, upon GAS6 stimulation. These discoveries should serve as a potentially useful resource for studying AXL functions as well as for the development of effective therapeutic options to target AXL.

**Abstract:**

Overexpression and amplification of AXL receptor tyrosine kinase (RTK) has been found in several hematologic and solid malignancies. Activation of AXL can enhance tumor-promoting processes such as cancer cell proliferation, migration, invasion and survival. Despite the important role of AXL in cancer development, a deep and quantitative mapping of its temporal dynamic signaling transduction has not yet been reported. Here, we used a TMT labeling-based quantitative proteomics approach to characterize the temporal dynamics of the phosphotyrosine proteome induced by AXL activation. We identified >1100 phosphotyrosine sites and observed a widespread upregulation of tyrosine phosphorylation induced by GAS6 stimulation. We also detected several tyrosine sites whose phosphorylation levels were reduced upon AXL activation. Gene set enrichment-based pathway analysis indicated the activation of several cancer-promoting and cell migration/invasion-related signaling pathways, including RAS, EGFR, focal adhesion, VEGFR and cytoskeletal rearrangement pathways. We also observed a rapid induction of phosphorylation of protein tyrosine phosphatases, including PTPN11 and PTPRA, upon GAS6 stimulation. The novel molecules downstream of AXL identified in this study along with the detailed global quantitative map elucidating the temporal dynamics of AXL activation should not only help understand the oncogenic role of AXL, but also aid in developing therapeutic options to effectively target AXL.

## 1. Introduction

Breast cancer has become the most common cancer, with more than 2.2 million cases in 2020 [1]. Breast cancer is a heterogeneous disease comprising four primary molecular subtypes, defined in large part by the expression status of hormone receptors (HR), including estrogen receptor (ER), progesterone receptor (PR) and HER2 receptor tyrosine kinase (RTK). Luminal A breast cancers are HR+/HER2− and account for ~50–60% of breast cancer cases. Luminal B breast cancers are HR+/HER2+ and account for ~10 percent, while HER2-enriched (HER2+) breast cancers account for about ~15–20% and triple negative breast cancer (HR-/HER2-) (TNBC) accounts for ~12% [2]. Although immune checkpoint blockage- and PARP inhibitor-based therapies have recently been approved for some TNBC patients [3,4,5,6], unlike the other three subtypes of breast cancer, targeted therapies are not available for most patients with TNBC. Some RTKs, including EGFR [7], c-MET [8,9] and IGF1R [10], have been reported to be upregulated in subsets of TNBCs. Our previous work using mass spectrometry-based quantitative phosphoproteomics to analyze a panel of TNBC cell lines led to the discovery that AXL was overexpressed and activated in ~40% of TNBC cell lines with high invasion and strong anti-anoikis oncogenic phenotypes [11]. We also showed that higher expression of AXL was significantly associated with poor prognosis of patients with TNBC and that a humanized anti-AXL monoclonal antibody could inhibit oncogenic phenotypes in vitro and reduce tumor formation in xenograft mouse models [11].

AXL is a member of the TAM family, which is composed of Tyro3, AXL, and Mer RTKs [12]. Although amplification and mutations are rare in AXL, overexpression of the AXL protein has been reported in many different cancers, including breast, ovarian, prostate, non-small cell lung cancer and head and neck squamous cell carcinoma [13]. AXL can be predominantly activated by the vitamin K-dependent growth arrest–specific 6 (GAS6) protein [14]. Although GAS6 binds to all TAM family members, it has the highest affinity for AXL [12]. Activation of AXL signaling has profound effects on promoting cell proliferation, migration and invasion, epithelial-mesenchymal transition (EMT) and tumor angiogenesis [15]. Recently, AXL has been discovered to be associated with multiple RTKs, such as EGFR [16], HER2 [17], ALK [18] and VEGFR [19], resulting in the activation of these RTKs in a ligand-independent manner, which induces resistance to the corresponding targeted therapies. As a core member of the TAM RTK family, AXL can also function as an important negative inflammatory mediator, suppressing dendritic cells, natural killer cells, and macrophages [20,21,22]. Activation of AXL signaling has an immunosuppressive role to reduce the antitumor immune response and help cancer cells avoid host immune surveillance [23,24].

Despite the multifunctional roles of AXL in cancer initiation and progression and in regulating antitumor immune responses, the signaling network driven by AXL has not been fully characterized. A quantitative global phosphoproteome survey focusing on the temporal dynamics of tyrosine phosphorylation signaling events driven by GAS6/AXL has not been performed. In this study, we employed a 16-plex tandem-mass tag (TMT) system [25] coupled with mass spectrometry-based proteomics to quantitatively map the tyrosine phosphorylation dynamics driven by the activation of AXL. Our studies identified dynamic changes of tyrosine phosphorylation not only during early phases (5 and 10 min) of AXL activation but also at later phases (1 and 5 h) of AXL activation. We observed a stepwise amplification of tyrosine signaling in the early and middle phases of the activation and a decrease of signaling in the late phase of the activation. In addition to elevation of tyrosine phosphorylation, we also observed a reduction in tyrosine phosphorylation of some proteins upon AXL activation. Our study demonstrates the complexity of AXL signaling regulation and provides novel insights into the signaling transduction driven by the activation of AXL.

## 2. Methods

### 2.1. Cell Culture and GAS6 Stimulation

MDA-MB-231 cells (ATCC) were cultured in DMEM with 10% (vol/vol) FBS and 1% penicillin/streptomycin in a 5% CO_2_ incubator at 37 °C. HCC1395 cells (ATCC) were cultured in RPMI-1640 with 10% (vol/vol) FBS and 1% penicillin/ streptomycin in a 5% CO_2_ incubator at 37 °C. Cells in tissue culture plates at 80% confluency were washed twice with 1X PBS and incubated for 18 h in serum-free media before GAS6 stimulation. Recombinant human GAS6 (R&D Systems, Minneapolis, MN, USA) (400 ng/mL) was directly added to serum-free media for 5 min, 10 min, 1 h and 5 h. An AXL inhibitor, 2 µM R428 (Selleckchem, Houston, TX, USA), was used to pretreat MDA-MB-231 cells for 30 min prior to GAS6 stimulation.

### 2.2. Western Blot Analysis

After stimulation, cells were harvested and lysed in modified RIPA buffer (50 mM Tris-HCl, pH 7.4, 150 mM NaCl, 1 mm EDTA, 1% Nonidet P-40, 0.25% sodium deoxycholate, and 1 mM sodium orthovanadate in the presence of protease inhibitors) and centrifuged to remove undissolved components. The supernatant was subjected to SDS-PAGE, transferred to nitrocellulose membranes, and probed with specific primary antibodies and horseradish peroxidase-conjugated secondary antibodies. The primary antibodies used in this study were as follows: AXL (8661), AXL pTyr702 (5724), AKT (9272), AKT pS473 (9271), PZR (9893), and phospho-PZR Y263 (8088) SHP2 pY580 (3751), SHP2 (3397), PTPRA (4481), CDK1 (77055), CDK1 pY15 (9111), EPHA2 (6997), EPHA2 pY772 (8244) and beta-actin (12620) that were purchased from Cell Signaling Technology (Danvers, MA, USA). PTPRA pY798 (ab169769) was purchased from Abcam (Waltham, MA, USA).

### 2.3. Cell Lysis and In-Solution Trypsin Digestion

After stimulation, cells were harvested in modified RIPA buffer (50 mM Tris-HCl, pH 7.4, 150 mM NaCl, 1 mm EDTA, 1% Nonidet P-40, 0.25% sodium deoxycholate, and 1 mM sodium orthovanadate in the presence of protease inhibitors) for Western blot analysis. 8 M urea buffer (20 mM HEPES pH 8.0, 8 M urea, 1 mM sodium orthovanadate, 2.5 mM sodium pyrophosphate, 1 mM β-glycerophosphate, and 5 mM sodium fluoride) was utilized for proteomic analysis.

Following cell culture and GAS6 stimulation, cells were harvested and lysed in 8 M urea buffer (8 M urea, 20 mM HEPES pH 8.0, 1 mM sodium orthovanadate, 2.5 mM sodium pyrophosphate, 1 mM β-glycerophosphate, and 5 mM sodium fluoride), sonicated, and subsequently cleared by centrifugation at 15,000× *g* at 4 °C for 20 min. Protein concentration of lysates was measured by BCA protein assay. 5 mg of protein lysates from each treatment condition was used for in-solution trypsin digestion. Briefly, the protein lysates were reduced with 5 mM dithiothreitol at 37 °C for 1 h and alkylated with 10 mM iodoacetamide at RT in the dark for 30 min. After reduction and alkylation, the protein lysates were diluted in 20 mM HEPES pH 8.0 to a final concentration <2 M urea and were subjected to in-solution protease digestion using TPCK-treated trypsin (Worthington Biochemical Corp. Lakewood, NJ, USA) on an orbital shaker overnight at room temperature. Peptide digests were acidified with 20% trifluoroacetic acid (TFA) to a final concentration of 1% TFA to quench the digestion reaction and subjected to centrifugation at 10,000× *g* at room temperature for 10 min. The resulting supernatants were desalted using SepPak C_18_ cartridge (Waters Corporation, Milford, MA, USA). Eluted peptides were lyophilized to dryness, and lyophilized peptides were stored at −80 °C prior to phosphotyrosine peptide enrichment.

### 2.4. Affinity Enrichment of Phosphotyrosine Peptides

For phosphotyrosine peptide enrichment, 3 mg per replicate of lyophilized tryptic peptides were used. Phosphotyrosine peptides were enriched using a phosphotyrosine peptide enrichment kit (PTM Scan, P-Tyr-1000; Cell Signaling Technology, Danvers, MA, USA). Immunoaffinity purification (IAP) of phosphopeptides was carried out as previously described [11]. Briefly, after lyophilization, 3 mg of peptide mixture was dissolved in 1.4 mL of IAP buffer (50 mM MOPS pH 7.2, 10 mM sodium phosphate, 50 mM NaCl) and subjected to centrifugation at 2000× *g* at room temperature for 5 min. Before IAP, P-Tyr-1000 beads were washed with IAP buffer twice at 4 °C, and the pH of the supernatant containing peptides was adjusted to 7.2 by adding 1 M Tris base. For IAP, the supernatant was incubated with P-Tyr-1000 beads at 4 °C for 60 min, and the beads were washed three times with IAP buffer and then twice with water. Peptides were eluted twice from beads by incubating the beads with 50 µL 0.1% TFA at room temperature.

### 2.5. TMT Labeling of Peptides

The enriched pTyr peptides eluted from P-Tyr-1000 beads were vacuum-dried, reconstituted in 50 µL 100 mM triethylammonium bicarbonate (TEABC) and mixed with 50 µg TMTpro reagent that was dissolved in 10 µL anhydrous acetonitrile. After 1 h incubation at RT, 10 μL of 5% hydroxylamine was added and incubated for 15 min at RT to quench the labeling reaction. Peptides labeled by different TMT reagents were then mixed and dried with Speed-Vac. Dried peptides were reconstituted with 0.1% trifluoroacetic acid (TFA), desalted with C_18_ stage tips and vacuum dried. The desalted TMT-labeled peptides were kept at −80 °C prior to LC-MS/MS analysis.

### 2.6. LC-MS/MS Analysis

The peptide fractions were loaded on a 2 cm trap column (Acclaim PepMap 100, C_18_, 5 µm particle size, 100 µm i.d. 100 Å pore size, Thermo Scientific, San Jose, CA, USA) using 0.1% formic acid with a flow rate of 10 µL/min for 5 min. The peptides were separated on a 50 cm analytical column (Acclaim PepMap 100, C_18_, 2 µm particle size, 75 µm i.d. 100 Å pore size, Thermo Scientific, San Jose, CA, USA) with a 135 min gradient from 3% to 40% acetonitrile in 0.1% formic acid at a flow rate of 300 nl/min. The spray voltage was set to 2.2 kV while capillary temperature was set to 275 °C. The samples were analyzed on an Orbitrap Eclipse mass spectrometer (Thermo Scientific, Bremen, Germany). The MS instrument was operated in data-dependent acquisition mode. A survey full scan MS (from 350–1700 *m*/*z*) was acquired in the Orbitrap with a resolution of 120,000 at *m*/*z* 200 and with a maximum AGC target value of 800,000 ions. The data-dependent MS/MS was carried out using the Top Speed method with a duty cycle of 3 s. Singly charged precursor ions were excluded, while precursor ions with charge states 2–5 were sequentially isolated to a target value of 200,000 ions and fragmented in the higher-energy collisional dissociation cell using 34% normalized collision energy (NCE). The maximum ion injection time for MS and MS/MS was set to 50 ms and 86 ms, respectively. Fragment ion spectra were detected in Orbitrap mass analyzer with a resolution of 50,000 at *m*/*z* 200. Dynamic exclusion was enabled for one event of fragmentation followed by exclusion of the precursor for the next 35 s within 7 ppm of the selected *m*/*z*. For all measurements with the Orbitrap detector, a lock-mass ion from ambient air (*m*/*z* 445.120025) was used for internal calibration [26].

### 2.7. Mass Spectrometry Data Analysis

The Proteome Discoverer software suite (v 2.5; Thermo Fisher Scientific, San Jose, CA, USA) was used for quantitation and database searches. The MS/MS data were searched using the SEQUEST search algorithm against a Human Uniport protein database (20,369 protein sequences, ver. 07242019) supplemented with frequently observed contaminants. Search parameters included trypsin as a protease with full specificity and a maximum of two allowed missed cleavages; carbamidomethylation of cysteine and TMTpro tag (+304.207 Da) on lysine residues or peptide N-terminus as a fixed modification; and oxidation at methionine, deamidation at asparagine and glutamine, and phosphorylation at serine/threonine and tyrosine as variable modifications. The precursor tolerance was set at 10 ppm while the fragment match tolerance was set to 0.02 Da. The PSMs, peptides and proteins were filtered at a 1% false discovery rate cut-off calculated using target-decoy database searches. The probability of an identified phosphorylation of specific Ser/Thr/Tyr residue on each identified phosphopeptide was determined from the PhosphoRS algorithm [27]. All mass spectrometry datasets acquired for this study were deposited to ProteomeXchange (http://proteomecentral.proteomexchange.org, accessed on 1 August 2021) [28] and are available with the accession number PXD025457.

### 2.8. Phosphotyrosine Data Analysis

TMT reporter ions were extracted and normalization was performed based on the average of total phosphopeptide intensity detected in each channel. The phosphosite quantitation was calculated from the summation of the intensity of phosphopeptides with phosphorylation on the same residues. The ratios between cell lines and treatment groups were calculated based on the average intensity of replicate channels, and Student’s t-test was performed for statistical analysis. We chose a 1.5-fold cut off with *p*-value < 0.05 to consider peptides as demonstrating increased phosphorylation and a 0.67-fold cut off with *p*-value < 0.05 for peptides to considere as demonstrating decreased phosphorylation. Pathway enrichment analysis was performed using DAVID [29], an integrated online functional annotation tool, and plotted using the ggpubr package in R. The bubble plot for phosphorylation changes in kinases and phosphatases and Fuzzy C-means clustering showing the dynamic regulation patterns of phosphosites were generated using ggplot and mfuzz packages in R, respectively.

## 3. Results

### 3.1. AXL Is Highly Expressed in Triple Negative Breast Cancer Cell Line

**Figure 1:**
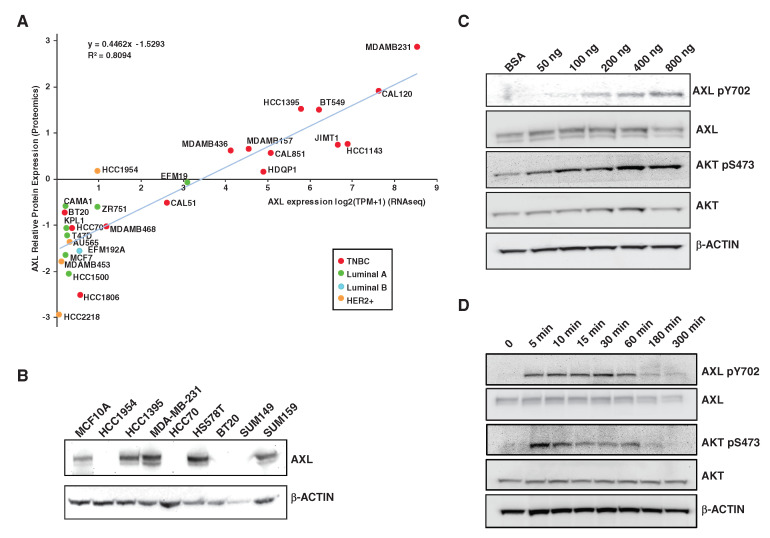
AXL expression and GAS6 stimulation in breast cancer cell lines. (**A**). Scatter plot showing AXL expression levels based on RNA-Seq data and its protein expression based on proteomics data (both from CCLA) as indicated. The color of the dots represents the subtype of breast cancer cell lines: TNBC (red), luminal A (green), luminal B (blue) and HER2+ (yellow). (**B**). Western blot results showing AXL expression level in a panel of nine TNBC cell lines. Beta-actin serves as a loading control. (**C**). MDA-MB-231 cells were treated with the indicated concentration (ng/mL) of GAS6 for 10 min. Phosphorylation levels of AXL and AKT and their total protein expression levels were examined by Western blot analysis. (**D**). MDA-MB-231 cells were treated with 400 ng/mL GAS6 for different time points as indicated. Phosphorylation levels of AXL and AKT and their total protein expression levels were examined by Western blot analysis as shown. (The original western blot images were included in Figure S1).

Overexpression of AXL receptor tyrosine kinase has been observed in multiple tumor types [12] and can induce epithelial-mesenchymal transition (EMT) and promote cancer cell proliferation, migration and invasion [15,16]. We previously performed tyrosine phosphoproteome profiling of a panel of triple negative breast cancer (TNBC) and discovered that AXL is hyperphosphorylated in highly aggressive TNBC cell lines [11]. A systematic investigation of AXL expression in a broader spectrum of breast cancer cell lines has not been performed. Using the RNA-Seq expression data and proteome data generated from Cancer Cell Line Encyclopedia (CCLE) [30,31], we plotted the expression level of AXL in 27 breast cancer cell lines composed of 15 TNBC, 4 HER+ and 8 ER+ luminal A and B breast cancer cell lines. This analysis clearly demonstrated that the AXL protein expression level is highly correlated with its mRNA expression levels (R^2^ = 0.81). The expression levels of AXL are extremely low in ER+ luminal and HER2+ breast cancer cell lines (Figure 1A). We performed immunoblotting to examine the expression levels of AXL in a subset of breast cancer cell lines including MCF10A, a spontaneously immortalized normal mammary gland epithelial cell line, HCC1954, a HER2+ breast cancer cell line and seven different TNBC cell lines (Figure 1B). We confirmed that four TNBC cell lines exhibited high expression of AXL (Figure 1B), with the highest expression in MDA-MB-231 in agreement with the analysis of CCLE data sets (Figure 1A). Thus, MDA-MB-231 cells were selected for the phosphoproteome study, because the high expression level of AXL in MDA-MB-231 cells would be likely to allow us to detect most of the signaling events driven by AXL activation.

To map the signaling pathways regulated by AXL activation, we first tested various doses of GAS6, the ligand of AXL, and monitored the AXL tyrosine 702 (Tyr702) phosphorylation that resides in the kinase domain of AXL and undergoes autophosphorylation. We found that 400 ng/mL GAS6 treatment strongly activates AXL and the downstream AKT pathway (Figure 1C). We next treated MDA-MB-231 cells with GAS6 for different amounts of time and checked phosphorylation of AXL and AKT. We found that AXL Tyr702 could be quickly phosphorylated in as few as 5 min, reached maximum phosphorylation at 30–60 min, and subsequently started to decrease. After 5 h treatment, AXL pTyr702 diminished to its basal level. We also observed that pAKT was upregulated and reached its highest level at 5 min of treatment, reduced after 10 min of treatment and diminished as low as the unstimulated level in cells with 5 h of treatment (Figure 1D).

### 3.2. Quantitative Profiling of Tyrosine Phosphoproteome Regulated by AXL in MDA-MB-231 Cells

**Figure 2:**
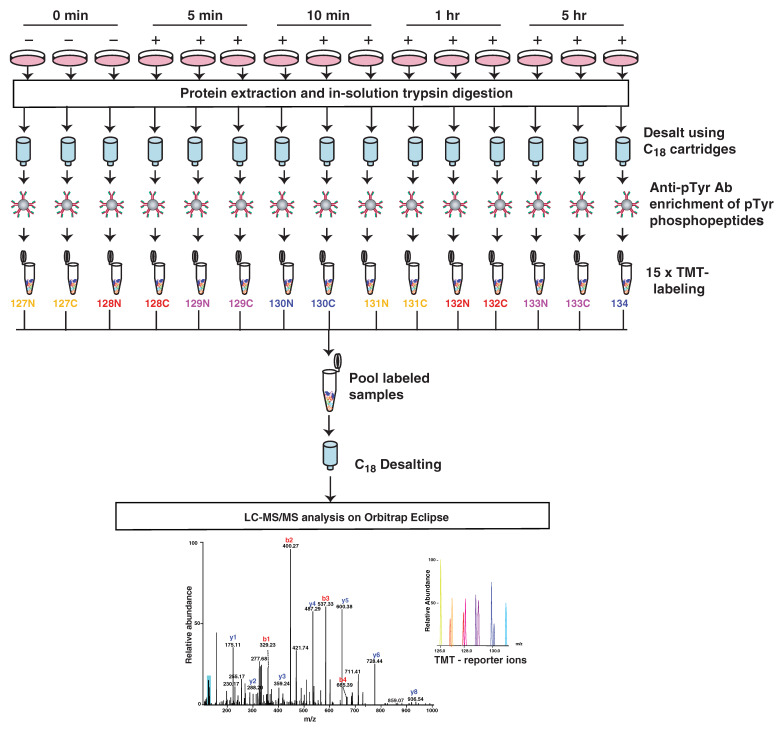
A schematic depicting the strategy used for temporal quantitative phosphotyrosine proteomic profiling of GAS6-treated MDA-MB-231 cells. All experiments were performed in triplicate. Cells were serum starved for 18 h and treated with 400 ng/mL GAS6 for 5 min, 10 min, 1 h or 5 h. After the GAS6 treatment, cells were harvested and lysed in 8 M urea buffer, followed by trypsin digestion and desalting. Tyrosine phosphorylated peptides were enriched with anti-pTyr antibody (pY1000). Enriched peptides were labeled with the TMTpro kit and analyzed by Orbitrap Eclipse.

In order to study the dynamic regulation of downstream signaling cascades by activation of AXL, we treated MDA-MB-231 cells with GAS6 for four different time points (5 min, 10 min, 1 h and 5 h). All treatments were performed in triplicate. After the treatment, cells were harvested in 8 M urea buffer, followed by trypsin digestion and C_18_-based reversed-phase desalting. Peptides with phosphorylated tyrosines were enriched using a pY1000 antibody mix that recognizes peptides containing phosphotyrosines. The enriched pTyr peptides were isobarically labeled using a 16-plex tandem mass tag (TMT) labeling system and analyzed using an Orbitrap Eclipse mass spectrometer (Figure 2).

**Figure 3:**
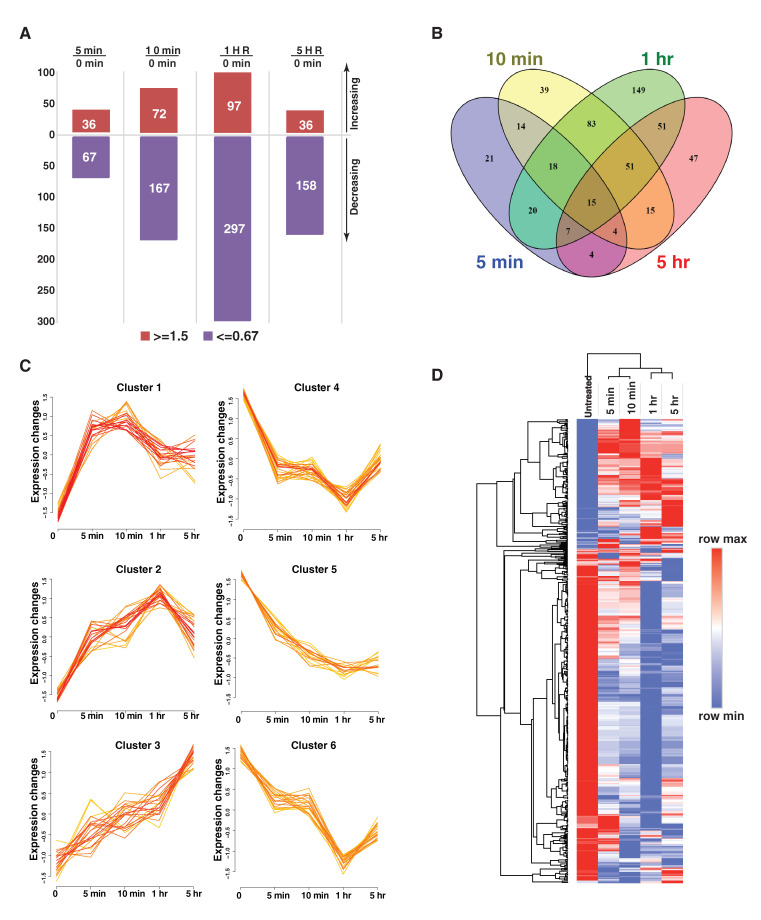
Dynamic regulation of tyrosine phosphorylation induced by the activation of GAS6/AXL signaling cascades. (**A**). Plot showing the number of upregulated and downregulated tyrosine phosphorylation sites at the indicated time points. (**B**). Venn diagram showing the overlap of regulated tyrosine phosphorylation sites at different times. (**C**). Fuzzy C-means clustering showing clusters of phosphotyrosine sites with dynamic regulation patterns. (**D**). A heatmap visualizing the hierarchical clustering of phosphotyrosine sites regulated by AXL. Each column represents the intensity at the indicated time points after GAS6 treatment.

In this quantitative tyrosine phosphoproteome study, we identified 1145 phosphotyrosine (pTyr) sites mapped to 721 proteins (Table S1). Among them, 538 pTyr sites from 383 proteins were altered (fold change >1.5, *p* value < 0.05) in at least one time point after GAS6 treatment. We observed a stepwise increase in the number of altered pTyr sites during GAS6 treatment from 5 min to 1 h, indicating the transduction of signal in the signaling network driven by the activation of AXL. Notably, at 5 h treatment, the number of altered pTyr sites dramatically decreased. We also observed that there were more pTyr sites whose phosphorylation levels were downregulated than those whose phosphorylation levels were upregulated (Figure 3A). This suggests that AXL could activate downstream protein tyrosine phosphatases that can potently reduce protein tyrosine phosphorylation and perhaps serve as a negative feedback system to balance the signaling events induced by the activation of AXL kinase.

We looked at the distribution of regulated pTyr sites at each time point after GAS6 stimulation. There are 15 pTyr sites that were commonly regulated at all time points (5 min, 10 min, 1 h and 5 h) (Figure 3B). AXL pY702 was consistently hyperphosphorylated after GAS6 stimulation. AXL pTyr 702 is one of the dominant autophosphorylation sites in the tyrosine kinase domain of AXL, and its phosphorylation leads to the activation of AXL kinase activity [32]. A highly conserved tyrosine site (Tyr38) that is shared with multiple histone H2B isoforms was consistently decreased in its phosphorylation levels after GAS6 stimulation. H2B Tyr38 has been recently reported to be phosphorylated by WEE1 [33], a nuclear tyrosine kinase that phosphorylates CDK1 Tyr15 and negatively regulates CDK1 activity to prevent mitotic entry before the completion of DNA synthesis [34]. H2B Tyr38 phosphorylation was found to repress histone cluster 1 (Hist1) transcription by excluding binding of the transcriptional coactivator NPAT and RNA polymerase II and recruiting the histone chaperone HIRA upstream of the Hist1 cluster [35]. Hypophosphorylation of H2B pTyr38 suggests that activation of AXL could have a role in promoting cell cycle progression. In addition, 52 and 188 pTyr sites were found uniquely regulated upon 5 min and 10 min of treatment, respectively, and 51 pTyr sites were commonly regulated upon 5 and 10 min’ treatments. These early-phase regulated tyrosine phosphorylation sites are critical for the initiation of AXL signaling. For instance, we observed an early upregulation of tyrosine phosphorylation of receptor tyrosine kinases of AXL, EPHB4 and EPHA2, non-receptor tyrosine kinases including ABL1/2, PTK2 (also known as FAK1), TNK2 and LYN, and multiple signaling adaptor proteins including GAB1, GAB2, ABI2, BCAR1, CAV1, DOK1 and NCK1. Several proteins involved in cytoskeleton rearrangement such as actin, TNS3, KIF23, CKAP2L, NEDD9 and PLEC (Figure 3B and Table S2) were also hyperphosphorylated, indicating the known regulatory role of AXL in promoting cancer cell migration/invasion and inducing epithelial-mesenchymal transition (EMT) [15].

We applied Fuzzy C-means clustering to these 538 regulated pTyr sites based on their phosphorylation levels and identified 6 clusters with different regulation patterns (Figure 3C). Cluster 1 represents pTyr sites that were upregulated in the early phase of the treatment (5–10 min) and started to reduce after 10 min. Cluster 2 represents the early upregulated pTyr sites which experienced a reduction of phosphorylation after 1 h treatment. Cluster 3 represents the pTyr sites that were continuously upregulated during the 5 h treatment. Clusters 4, 5 and 6 represent downregulated pTyr sites. The supervised clustering analysis for these 538 pTyr sites depicted similar patterns (Figure 3D). These analyses demonstrated the dynamic temporal regulation of the GAS6-AXL signaling network.

### 3.3. Activation of AXL Regulates Diverse Downstream Signaling Pathways

**Figure 4:**
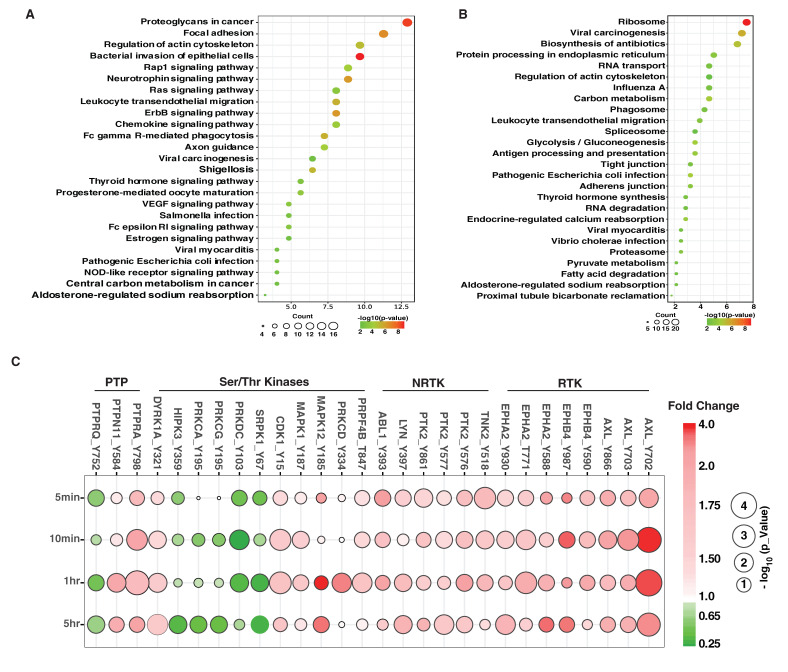
Signaling pathways activated by AXL. (**A**,**B**). Enriched signaling pathways for proteins with upregulated (**A**) or downregulated (**B**) tyrosine phosphorylation. Pathway analysis was performed using DAVID [29] based on the KEGG [42] pathway database and replotted using an R package. The size of the circle represents the number of proteins identified in each pathway. The color scheme represents −log_10_(*p*-value) as indicated. (**C**). Bubble plot displaying the alterations in tyrosine phosphorylation of protein tyrosine phosphatases (PTP), serine/threonine kinases, non-receptor tyrosine kinases (NRTK) and receptor tyrosine kinases (RTK). The color scheme represents the fold-changes upon GAS6 treatment of individual tyrosine phosphosites at the indicated time points. The size of the circle represents −log_10_(*p*-value) compared to untreated MDA-MB-231 cells.

In order to understand the signaling pathways regulated by AXL, we performed gene set enrichment analysis (GSEA) for proteins containing tyrosine phosphorylation sites that are upregulated (in Clusters 1, 2 and 3) (Figure 3C) or downregulated (in Clusters, 4, 5 and 6) (Figure 3C) upon GAS6 addition. Among the enriched gene sets that were upregulated due to the activation of AXL, we found several classical signaling pathways driven by receptor tyrosine kinases (RTK) and non-receptor tyrosine kinases (NRTK), including ERBB, VEGFR, focal adhesion, and RAS signaling pathways (Figure 4A). Multiple signaling pathways involved in cell adhesion, cell-cell junctions and cytoskeleton rearrangement were also regulated by the activation of AXL (Figure 4A,B), suggesting activation of AXL could regulate cancer cell migration and invasion.

Of note, several microbial infection-related pathways, including bacterial invasion of epithelial cells, viral carcinogenesis, viral myocarditis, pathogenic *E. coli* infection, salmonella infection and vibrio cholerae infection pathways, were also enriched after AXL was activated (Figure 4A,B). In this regard, it is interesting to note that AXL has been reported to be a receptor mediating the infection of multiple virus, such as West Nile virus, Zika virus and SARS-CoV-2 [36,37,38,39]. Thus, our findings suggest that the AXL signaling pathway could play an important role in regulating host responses to these viral infections. AXL was also found to be activated and involved in bacterial infection-mediated inflammatory responses [40,41].

### 3.4. Dynamic Regulation of Protein Kinases and Phosphatases

In this study, we employed a quantitative temporal phosphoproteomics analysis to resolve dynamic regulation of AXL signaling at the phosphosite level and reveal signaling outcomes that are directly connected to responsible upstream or downstream events. The phosphorylation of receptor molecules not only activates various signaling cascades but also deactivates the processes in later stages. In all, four receptor tyrosine kinases (RTK), four non-receptor tyrosine kinases (NRTK), ten serine/threonine kinases (STK) and three protein tyrosine phosphatases (PTP) were identified whose phosphorylation levels were significantly regulated by the activation of AXL. It is noteworthy that almost all tyrosine kinases, including RTKs and NRTKs, were rapidly hyperphosphorylated shortly (5 and 10 min) after GAS stimulation (Figure 4C), indicating the transduction of signals from extracellular signaling molecules to cell-surface receptors, triggering intracellular pathways that ultimately modulate cellular metabolism, function, or development. It is striking that the majority of tyrosine phosphorylation sites on serine/threonine kinases (STK) occurred during an intermediate or late phase (1 h and 5 h) after GAS6 stimulation. Further, in contrast to tyrosine kinases, almost half of the regulated sites in STKs were hypophosphorylated upon GAS6 addition (Figure 4C). We also observed increased phosphorylation of tyrosine residues on protein tyrosine phosphatases (PTPRA and PTPN11) and decreased phosphorylation of PTPRQ (Figure 4C). These inducible events can potentially modulate the activity of these tyrosine phosphatases, which could perhaps mediate the decrease of tyrosine phosphorylation of some of the sites that was observed (Figure 3A,C).

**Figure 5:**
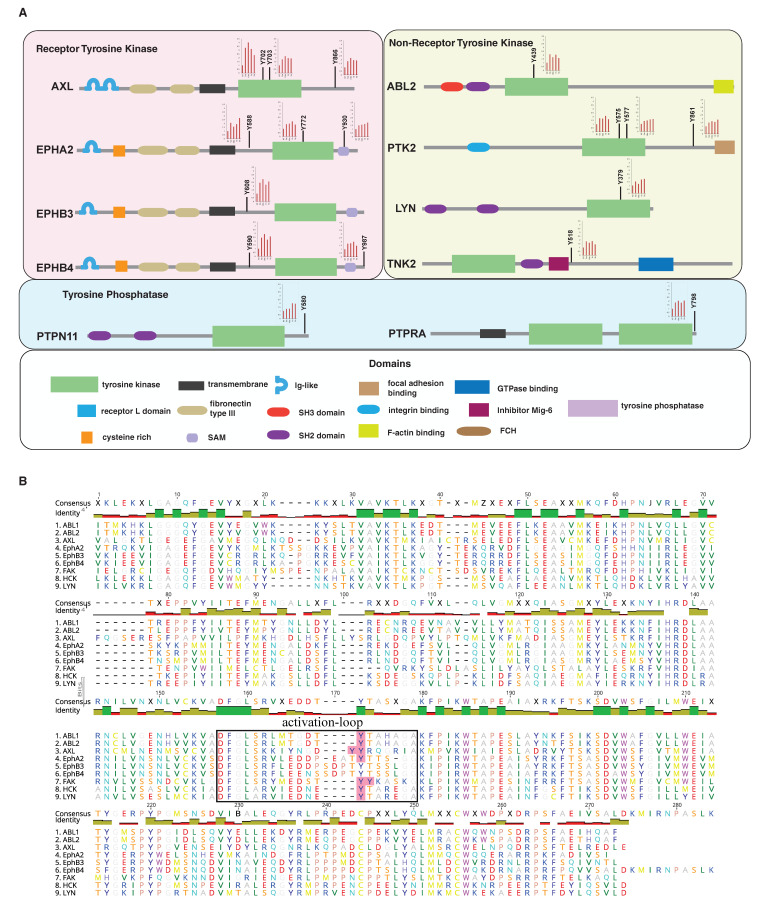
Site-specific regulation of tyrosine kinases and protein tyrosine phosphatases by AXL activation. (**A**). Schematic representation of receptor tyrosine kinases (top left panel), non-receptor tyrosine kinases (top right panel) and protein tyrosine phosphatases (middle panel) along with phosphorylation sites regulated by AXL activation and the time course. Black bars with red filled circles at the end denote phosphorylation modification. The embedded column plots depict the regulation pattern of individual phosphotyrosine sites. The domain structures of the molecules are shown with the key in the panel at the bottom. (**B**). Multiple sequences alignment of the amino acid sequences of the tyrosine kinase domain of the RTKs and NTRKs regulated by Axl. The box indicates the activation loop of the kinase domain. Regulated phosphotyrosine residues identified in this study were highlighted in pink.

To better understand the role of RTKs, NRTKs and PTPs in GAS-AXL signaling transduction, we mapped the upregulated phosphosites onto the domain structures of these proteins (Figure 5A). RTKs are composed of a large extracellular domain binding to ligands, a transmembrane domain and a cytoplasmic tail containing the tyrosine kinase domain. NRTKs typically comprise a tyrosine kinase domain along with other domains such as SH2 and SH3 domains that regulate catalytic activities and mediate interactions with other proteins. A number of the tyrosine phosphorylation sites that were found to be regulated were located within the kinase domains of RTKs and NRTKs. These sites have important roles in modulating the activity of the corresponding kinases. When we aligned the kinase domain sequences of tyrosine kinases that were regulated by AXL and marked regulated pTyr sites, we found that AXL pTyr702 and pTyr703, EPHA2 pTyr772, ABL1 pTyr393, ABL2 pTyr439, PTK2 (FAK1) pTyr576 and pTyr577, LYN pTyr397 and NCK pTyr411 were all located in the activation loop of the kinase domain whose phosphorylation levels are known to correlate with increased kinase activity [43] (Figure 5B).

We also found rapid induction of phosphorylation at Tyr866 in AXL (Figure 5A)—this site is autophosphorylated when AXL is activated. Phosphorylation of AXL at Tyr866 leads to subsequent binding to SRC, LYK and PLC and modulation of downstream signaling pathways, including the PI3K/AKT/mTOR, JAK/STAT, NF-κB, and RAS/RAF/MEK/ERK pathways [44]. Several Eph family receptors were also rapidly hyperphosphorylated by AXL (Figure 5A). In addition to the tyrosine sites in the activation-loop of tyrosine kinase domain, EPHA2 pY588 and pY960, EPHB4 pTyr590 and pTyr987 and EPHB3 pTyr608 were also upregulated during the early phase of AXL activation. Tyrosine 588 is located in the juxtamembrane region of EphA2 and is implicated in regulation of the kinase activity and in providing a binding site for Vav GEFs [45,46]. While pY960 is in the sterile alpha motif (SAM) domain of EPHA2, phosphorylation at Tyr930 enables differential binding to the Src homology 2 domain of the adaptor protein Grb7, regulating signal transduction [47]. Tyr987 is at the c-terminal tail end of EPHB4 and was shown to be phosphorylated by insulin receptor isoform-A kinase-associated activity in response to autocrine IGF-II stimuli. Tyr987 is close to a ubiquitin binding motif (958–973), and phosphorylation of Tyr987 can reduce ubiquitination level of EPHB4 and increase its stability [48]. Overall, these data clearly indicate that activation of AXL can induce phosphorylation of, and cross-activation of, EPH receptors.

As common signaling regulators downstream of RTKs, NRTKs are often directly/indirectly phosphorylated by activated RTKs. In our study, we observed that several NRTKs, including PTK2 (FAK1), ABL1/2, TNK2 and LYN, were hyperphosphorylated by GAS6 stimulation (Figure 5A). AXL was recently reported to have bidirectional regulatory roles with ABL2 [49,50]. Activation of AXL by GAS6 can induce interaction between ABL2 and AXL. Suppressing ABL2 with inhibitors or siRNA can reduce AXL tyrosine phosphorylation. On the other hand, the knock-down of AXL can also reduce ABL2 phosphorylation [50]. In our study, we detected that GAS6-dependent AXL activation can also induce ABL2 phosphorylation on pTyr439. TNK2 (also known as ACK1) is an intermediary kinase that rallies signals from RTKs to intracellular signaling networks. Multiple ligands such as EGF, heregulin, IGF and GAS6 can induce phosphorylation of TNK2, suggesting that TNK2 acts as a major integrator of RTK signaling [51]. Our previous work revealed that AXL and TNK2 are both overexpressed in highly aggressive TNBC cells [11]. In this study, we found that activation of AXL can induce a rapid temporal increase in phosphorylation of TNK2 pTyr518, which is located downstream of a Cdc42- and Rac-interactive binding (CRIB) domain; phosphorylation of Tyr518 mediates the interaction between TNK2 and the SH2 and SH3 domains of SRC kinase [52].

In addition to tyrosine kinases, we also found altered phosphorylation levels of several protein tyrosine phosphatases, including PTPRA, PTPN11 and PTPRQ (Figure 5A). Phosphorylation of C-terminal tyrosine (Tyr798) of PTPRA has been shown to be associated with its phosphatase activity and is required for the interaction with the GRB2 SH2 domain [53]. pTyr 798 is also essential for dephosphorylation of Src at Tyr527 [54]. PTPN11 (also known as SHP2) is a non-receptor protein tyrosine phosphatase. It is involved in numerous signaling events downstream of receptors for growth factors, including interactions with cytokines, hormones, and extracellular matrices in the control of cell growth, differentiation, migration, and death [55]. PTPN11 pTyr580 was observed in our study to be gradually upregulated during the stimulation of GAS6 (Figure 5A). pTyr 580 is in the C-terminus of PTPN11 and can be elevated by the activation of multiple RTKs [56]. Phosphorylation on Tyr580 and Tyr542 is thought to relieve basal inhibition and stimulate PTPN11 tyrosine phosphatase activity [57]. PTPN11 Tyr542 has been shown to be phosphorylated by PDGFR-b [56], and ABL, a NRTK, was recently shown to phosphorylate Tyr580 [58], which induces intramolecular binding of pTyr580 to the C-SH2 domain and activates its phosphatase activity [59]. The activation of protein tyrosine phosphatases may explain the wide spectrum of dephosphorylation events that we observed after the activation of the GAS6-AXL signaling cascade (Figure 3A).

### 3.5. Validation of the Phosphotyrosine Profiling by Western Blot Analysis

**Figure 6:**
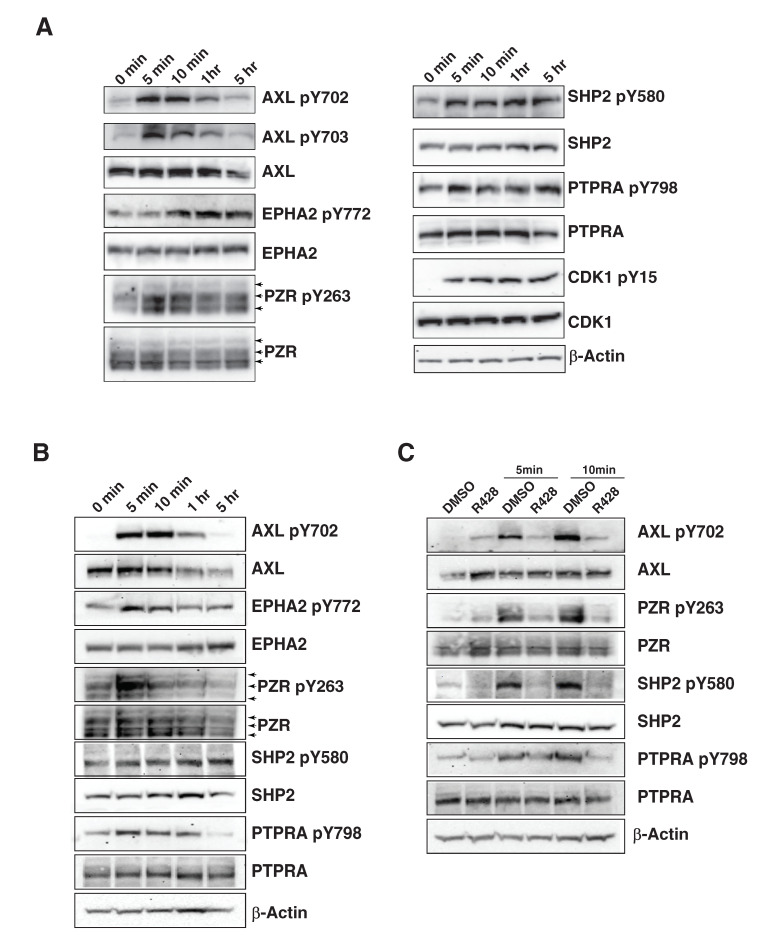
Western blot-based validation of selected molecules in the Axl signaling pathway. (**A**). MDA-MB-231 cells were treated with 400 ng/mL GAS6 for the indicated duration. Cell lysates were analyzed with Western blot analysis using phospho-specific antibodies against EPHA2 (pY772), AXL (pY703/pY702), PZR (pY263), SHP2/PTPN11 (pY580), PTPRA (pY798) and CDK1 (pY15) and antibodies against the corresponding protein as indicated. β-actin served as a loading control. The arrows indicate the three isoforms of PZR protein. (**B**). Western blot analysis to confirm the dynamic regulation of phosphorylation of the indicated proteins in HCC1395 cells treated with 400 ng/mL GAS6 for different time courses (as indicated). (**C**). MDA-MB-231 cells were pretreated with 2 µM R428 prior to GAS6 stimulation for 5 or 10 min, respectively. Protein phosphorylation levels were detected by indicated phospho-specific antibodies. (The original western blot images were included in Figure S1).

To validate our discoveries, we performed Western blot analysis using antibodies against phosphorylation sites of selected proteins, EPHA2, PTNP11, PTPRA, PZR and CDK1, that were identified from the global phosphotyrosine profiling. For each of these molecules, Western blot analysis confirmed that the levels of phosphorylation were consistent with the mass spectrometry results (Figure 6A). We treated MDA-MB-231 cells with 400 ng/mL GAS6 for the same time course as we performed for the tyrosine phosphoproteomic study. We first validated the activation of AXL by GAS6 and confirmed that AXL Tyr702 and Tyr703 were capable of being rapidly autophosphorylated in 5 min after GAS6 stimulation, achieved the maximum phosphorylation at 10 min and started to diminish around 1 h. Five hours after treatment, the phosphorylation levels returned to the untreated levels. We also noticed a reduction of total AXL protein levels after 5 h’ stimulation of GAS6, which is regulated by endocytosis of activated RTKs [60]. We then confirmed the phosphorylation changes of PTNP11 on Y560 and PTPRA on Y798 (Figure 6A). We also performed Western blot analysis to examine the phosphorylation change of P zero-related protein (PZR), also known as MPZL1, which is a cell surface transmembrane protein belonging to the immunoglobulin family and of which there are three isoforms, namely PZR, PZRa, and PZRb [61]. The intracellular part of PZR contains two immunoreceptor tyrosine-based inhibition motifs (ITIMs) which bind to tyrosine phosphatase PTPN11 [62]. Tyr263 is located within the ITIM motif, and its phosphorylation enhances the interaction between PTPN11 and PZR. Unlike phosphorylation of AXL, the observed increase of phosphorylation of PZR, PTPN11 and PTPRA did not diminish after 5 h stimulation. An interesting discovery of this study is a rapid activation of EPH receptor kinases, including EPHA2, EPHB4 and EPHB3. AXL has been shown to cross-phosphorylate multiple RTKs, including EGFR, HER2, FGFR, MET and ALK [12,13], and induce ligand-independent activation of these RTKs, which is associated with resistance to multiple RTK inhibitors. Our Western blot validation also confirmed that AXL activation can rapidly phosphorylate EPHA2 (Figure 6A). Our validation studies demonstrated that the total protein for each of these proteins (except AXL) did not change due to activation of AXL. This indicates that these proteins are hyperphosphorylated through activation of kinases and not because of transcriptional/translational regulation. To further validate the results from our phosphoproteomic analysis, we subjected HCC1395 TNBC cells, which express relatively high levels of AXL, to GAS6. We observed a similar dynamic regulation of phosphorylation of signaling proteins in the AXL signaling pathway in HCC1395 cells (Figure 6B). To confirm that the alteration in phosphorylation of these downstream proteins is dependent on AXL kinase activity, we pretreated MDA-MB-231 cells with 2 µM R428 for 30 min prior to GAS6 stimulation. R428 is a potent AXL-specific inhibitor that has 50- and >100-fold higher selectivity for AXL as compared to the other two TAM family kinases, Mer and Tyro3, respectively [63]. Figure 6C shows that short-term inhibition of AXL can not only suppress AXL autophosphorylation stimulated by GAS6, but can also substantially suppress the tyrosine phosphorylation of AXL downstream signaling molecules discovered in this study (Figure 6C).

## 4. Conclusions

Through TMT labeling-based quantitative phosphoproteomic profiling, we mapped the temporal dynamics of global tyrosine phosphorylation regulated by the activation of AXL signaling. Our study revealed that AXL activation could upregulate tyrosine phosphorylation of a widespread range of signaling proteins, but also could induce downregulation of phosphorylation of a broad spectrum of tyrosine sites. Activation of AXL by GAS6 can rapidly induce tyrosine phosphorylation and activation of multiple EPH family receptors, including EPHA2, EPHB3 and EPHB4, as well as several downstream non-receptor tyrosine kinases. In addition to the activation of tyrosine kinases, we also found that protein tyrosine phosphatases such as SHP2/PTPN11 and PTPRA that play pivotal roles in regulating signal transductions can also be hyperphosphorylated due to the activation of GAS6. Multiple signaling pathways involved in cancer initiation and progression, including the EGFR, VEGFR, focal adhesion and RAS signaling pathways, can be rapidly activated after the activation of AXL signaling. Overall, our study provides a detailed landscape of dynamic regulation of tyrosine phosphorylation driven by AXL activation, which demonstrates the complexity of AXL signaling and reveals the role of AXL in modulating signaling networks to promote breast cancer progression. These discoveries should serve as a potentially useful resource for studying AXL functions as well as for the development of effective therapeutic options to target AXL.

## Data Availability

Raw mass spectrometry files used in this experiment have been uploaded to the ProteomeXchange Consortium (http://proteomecentral.proteomexchange.org, accessed on 1 August 2021, via the PRIDE partner repository with the dataset identifier PXD025457.

## Supplementary Materials

The following are available online at https://www.mdpi.com/article/10.3390/cancers13164234/s1, Figure S1: original Western Blot, Table S1: A list of tyrosine phosphorylation sites detected by mass spectrometry-based phosphoproteomic analysis.
