# Supplementary material for: Quantitative Tyrosine Phosphoproteome Profiling of AXL Receptor Tyrosine Kinase Signaling Network
Source: Cancers (Basel). 2021 Aug 23;13(16):4234. doi: 10.3390/cancers13164234 (PMC8394654; doi:10.3390/cancers13164234)
Supplement: Supplementary file 1 [file cancers-13-04234-s001.zip › cancers-1334473-supplementary figure.pdf]

cancers

MDPI

Supplementary Material: Quantitative Tyrosine Phosphoproteome Profiling of AXL Receptor Tyrosine Kinase Signaling Network

Xinyan Wu, Li Wang, Nicole A. Pearson, Santosh Renuse, Ran Cheng, Ye Liang, Dong-Gi Mun, Anil K. Madugundu, Yaoyu Xu, Parkash S. Gill and Akhilesh Pandey

Figure 1B

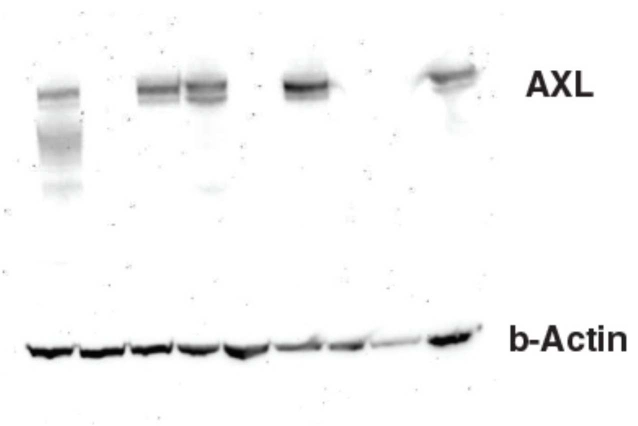

Figure 1C

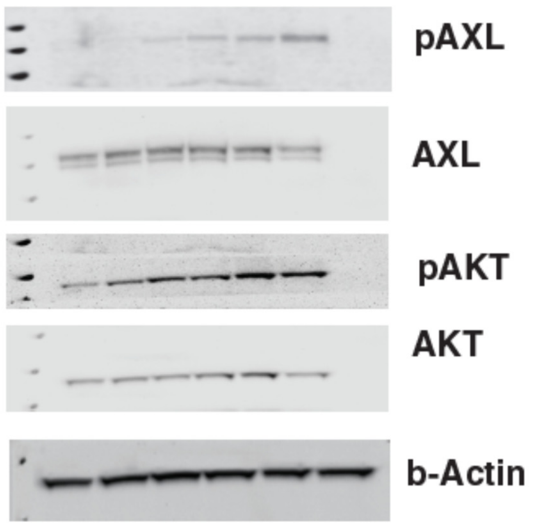

Figure 1D

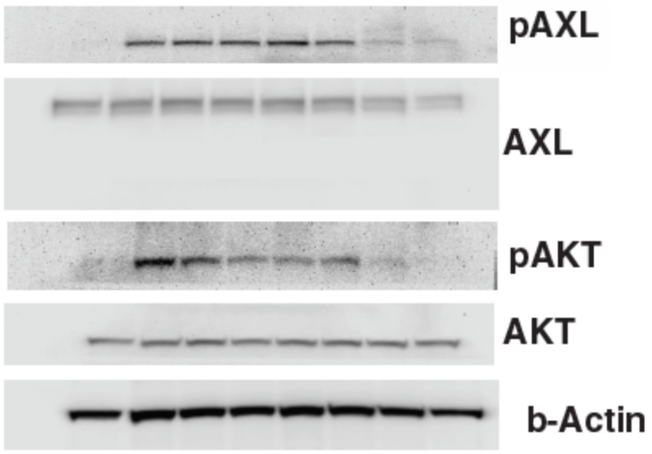

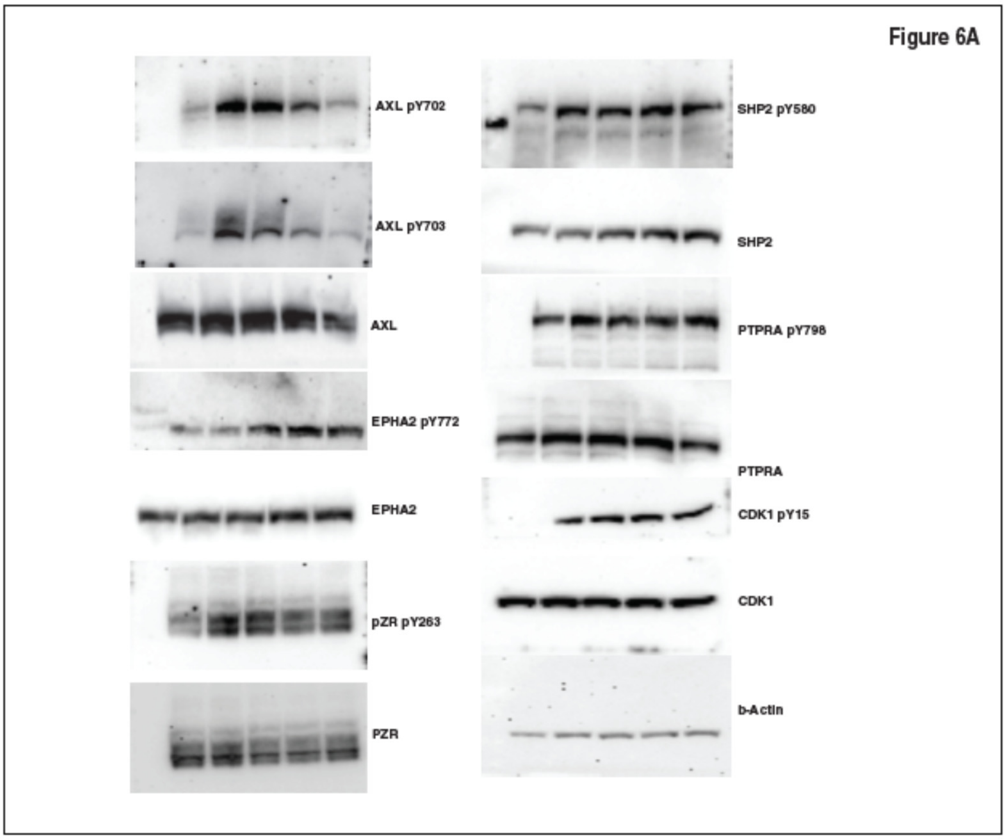

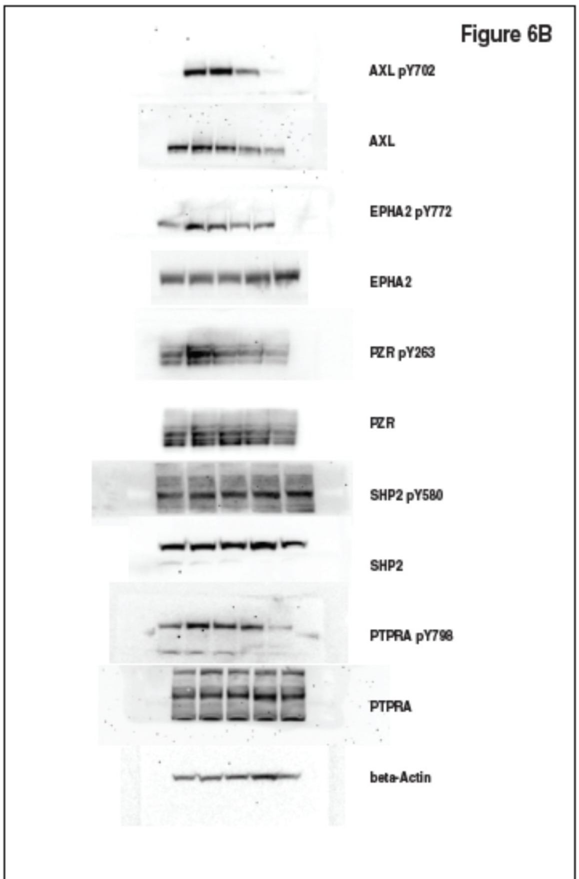

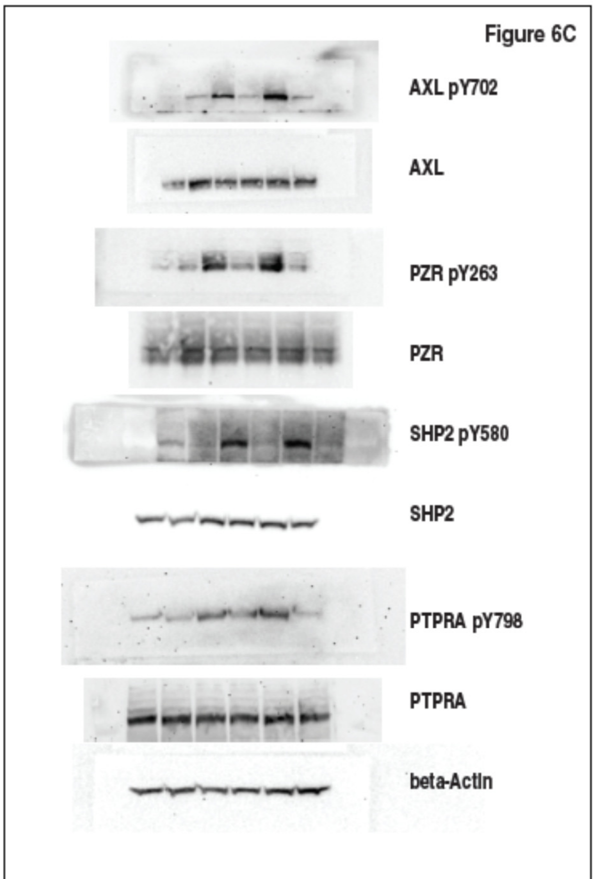

Figure S1. Original Western Blot.
